# Intestinal Intussusception: A Shocking Diagnosis

**DOI:** 10.7759/cureus.25368

**Published:** 2022-05-26

**Authors:** Sara M Morais, Cristina Santos Costa, Maria B Mourato, Tamiris Mogne, Guilherme Santos

**Affiliations:** 1 General Surgery, Unidade Local de Saúde do Norte Alentejano, Portalegre, PRT

**Keywords:** small intestine, surgery, idiopathic, adult, intussusception

## Abstract

Intussusception is a rare condition diagnosed in adults, with few cases reported as idiopathic. It is defined as the invagination of an intestinal segment into another adjacent one, due to the presence of a lead point, or in some cases, without identifiable causative lesions. The presentation is non-specific, even with careful evaluation, and most of the time, the diagnosis is made during surgery. We hereby present the case of a 73-year-old woman with idiopathic intussusception who presented in the emergency room. She was taken to the operating theatre, where intestinal resection was performed. Few cases of true idiopathic intestinal intussusception in adults are seen, and literature on this topic remains scarce. We discuss diagnostic and therapeutic options and do a brief review of the literature.

## Introduction

Intussusception in the adult population is a rare cause of abdominal pain. It is a challenging diagnosis, resulting in important morbidity and mortality due to delayed diagnosis [1]. The first described case was reported in 1674 by Paul Barbette, but throughout history, there have been other reports of intussusceptions [2,3].

It is a common pathology in children, representing the second most common cause of acute abdominal pain after appendicitis [4], but the incidence in the adult population is rarer, with incidence reported up to 5-10% of all cases of intussusception [1,3] comprising a merely 1-5% of all causes of intestinal obstruction [3,5].

Intestinal intussusception is the result of an abnormal peristaltic movement, leading to the "telescoping" of an intestinal segment into another adjacent segment [5]. The proximal part is designated the intussusceptum, and the distal part is known as the intussuscipiens [4]. Although the predominant etiology in the pediatric population is idiopathic, in adults, this phenomenon is typically due to a pathologic alteration, a lead point, which alters the normal peristalsis of the intestine and predisposes it to the advancement of the proximal intestine into the lumen of a fixed distal intestinal segment [3]. Of note, up to 57% of reported cases can be due to a malignant tumor [4], hence the importance of the recognition of this situation.

According to the literature, in 90% of adult intussusception cases, a pathologic cause is identified [2], such as a tumor, pancreas divisum, polyps, a Meckel diverticulum, or mesenteric cysts [5], among others.

In 1956, Dean et al. proposed a classification system for adult bowel intussusception which categorized cases into four distinct anatomic variants [4]: entero-enteric, affecting only the small intestine; colo-colic, involving the large bowel; ileocecal, when the ileocecal valve is the lead point; and ileocolic, when the terminal ileum prolapses to the ascending colon through the ileocecal valve [4,5]. Another classification system categorizes cases based on the etiology of the intussusception, benign or malignant. Furthermore, classification can also be made based on the presence of a leading point [4].

Any segment of the gastrointestinal tract can be involved [4], but there are some portions where a more freely mobile segment can go through a fixed one, due to adhesions or for its retroperitoneal location [4]. Therefore, most of these intussusceptions [2] tend to occur within the bowel (small or large). The ileum is the most frequent location (ileoileal intussusceptions are the most common, and double ileoileal or ileocecal are the less frequent), and only roughly 10% occur in extraintestinal locations [2].

Abdominal pain is the most common presenting symptom, followed by changes in regular bowel habits, nausea, vomiting, and gastrointestinal bleeding [6]. If not promptly diagnosed, intestinal intussusception may result in serious complications such as bowel obstruction or even strangulation and ischemia [2].

## Case presentation

**Figure 1:**
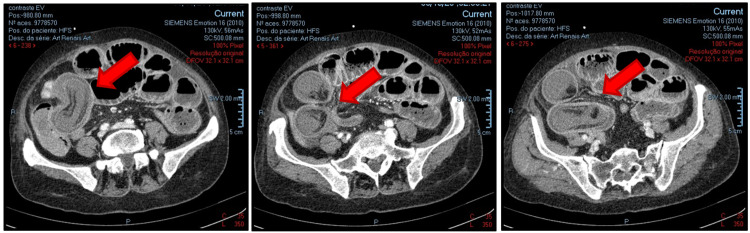
CT scan with typical findings of bowel within bowel, suggesting intussusception (axial view)

**Figure 2:**
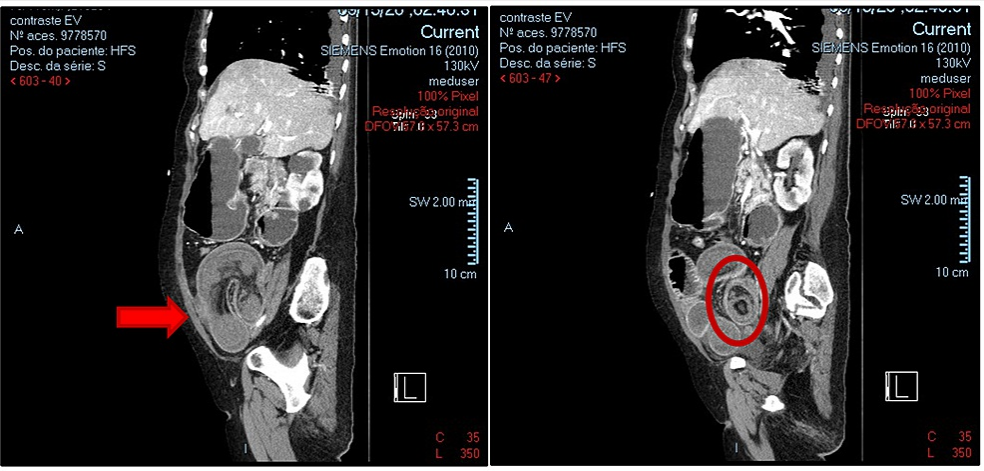
CT scan with typical findings of bowel inside bowel, suggesting intussusception (sagittal views) with special note to the target sign appearance on the image on the right

A 73-year-old woman with no relevant medical history was admitted to the emergency room with a shock of unknown cause and multiorgan failure (hyperlacticaemia with acidosis - lactate 7.5mmol/L, pH 7.39, hypotension, acute kidney injury AKIN3, altered mental status). On examination, the abdomen was distended and tender to palpation. She was promptly resuscitated and exhibited some overall improvement. A nasogastric tube was inserted, draining approximately 500mL of enteric fluid. As her mental status improved, she was unsure why she came to the emergency room but was able to report a two-month history of constipation and non-specific weight loss. She denied any previous abdominal pain, vomiting, or gastrointestinal bleeding. Laboratory analysis revealed normal hemoglobin and no leukocytosis, but did demonstrate an elevated C-reactive protein (CRP, 50.5 mg/L), acute renal failure (creatinine: 3.10 mg/dL), and elevated amylasaemia (435 U/L). A computerized tomography scan (CT) was done, revealing evidence of intussusception in the right iliac fossa (Figure 1, 2).

**Figure 3:**
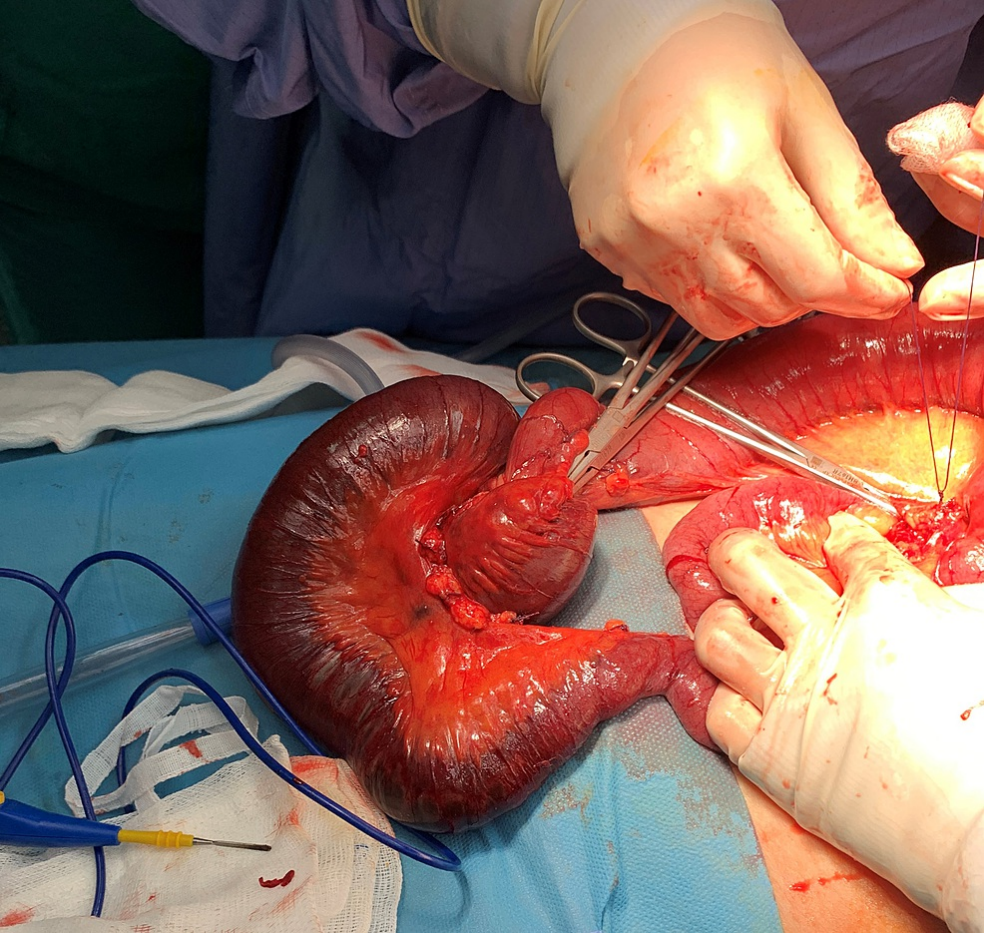
Intraoperative findings showing an enteroenteric intussusception

**Figure 4:**
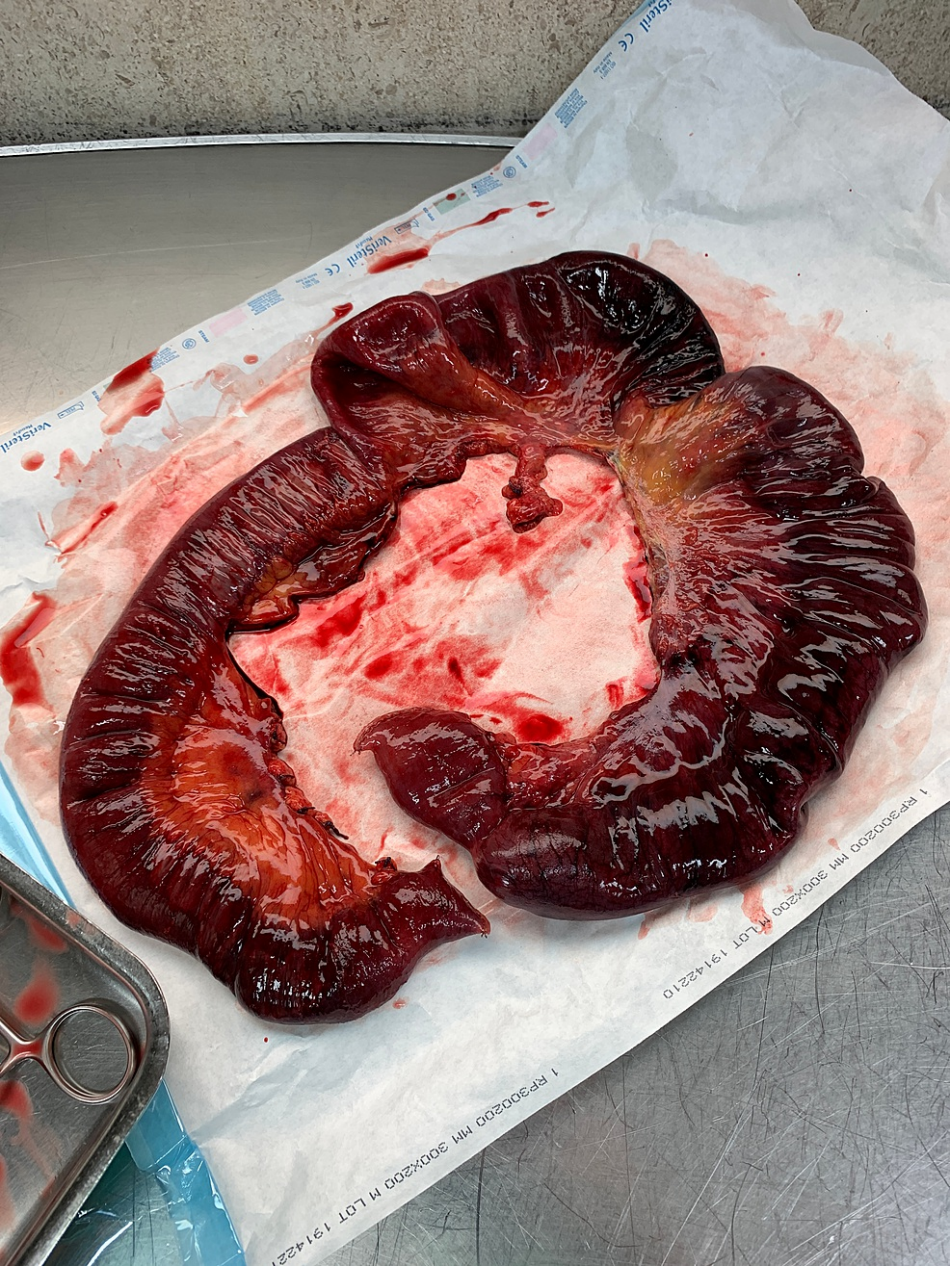
Resected ischemic small bowel, without identifiable lead point lesions

The patient was taken to the operating theatre, a laparotomy was performed, and a small bowel intussusception was confirmed with evidence of irreversible ischemia (Figure 3). It was decided to resect the affected segment of the bowel without attempting reduction. No anastomosis was done due to the patient’s marked hemodynamic instability during surgery. An end ileostomy and mucous fistula were fashioned. Further evaluation of the resected small bowel (approx. 110 cm) revealed no evidence of a pathological lead point (Figure 4). Pathological evaluation of the resected segment revealed only ischemic enteritis.

The patient was planned for ileostomy closure following stabilization, but on postoperative day 25, the patient suffered a cardiorespiratory arrest. Return of spontaneous circulation was achieved after resuscitation and advanced life support measures were taken; however her clinical course continued to decline, and she eventually died on postoperative day 44.

## Discussion

Intussusception is a rare diagnosis in the setting of abdominal pain or bowel obstruction in the surgical patient [5]. It accounts for only 1% of adults presenting with bowel obstruction [6,7]. In children, intussusception is often idiopathic, meaning an etiology is not identified. However, in adults, a lead point is found in around 80-90% of cases, even though the annual incidence of adult intussusception is very low [2]. The remaining of these patients have primary or idiopathic intussusception [6]. Large bowel intussusception is secondary to malignancy in about 65-70% of cases, but in small bowel intussusception, a benign lead point is a more frequent cause, with malignancy presented in only 30-35% of cases [3,5,6]. According to various sources, roughly 8-20% of all cases of small bowel intussusception are idiopathic in nature, though it is suspected other pathologies, including abnormal peristalsis or thyroid abnormalities, may contribute to its occurrence [1,2,8].

Abdominal computed tomography (CT) is the most sensitive diagnostic scan for intussusception in adults, with an accuracy of 58-100% [1]. Typical features include a "target" sign (representing a triangular adipose density of the mesentery of the intussusceptum) and a sausage-like lesion (representing alternating layers of low and high attenuation of the mesenteric fat and bowel wall, demonstrating advanced stages) [1]. In patients presenting following a prolonged period of intussusception, ischemia may occur, and a reniform mass may be seen on CT as a result of edema and necrosis. A CT scan also aids in cases of suspected malignancy and helps provide staging data [1].

Intussusception in adult patients has been classified in the literature according to location or presence of malignancy. In many cases, the lead point is a benign tumor, such as lipoma or adenomyoma [3], but some studies report malignant lead points in up to 50% of cases, most commonly adenocarcinoma [5]. Malignant causes of small bowel intussusception include other tumors such as leiomyosarcomas, adenocarcinoma, gastrointestinal stromal tumors (GIST), carcinoid and neuroendocrine tumors, or lymphomas. Due to the high risk of an underlying malignancy, surgery is recommended for adult patients [5]. Anemia and colonic intussusception have been reported as independent predictors of malignancy [6]. Other less frequent causes of intussusception include infections, postoperative adhesions, Chron's granulomas, Meckel's diverticulum, or other focal lesions such as sarcomas or lymphomas. The preoperative probability of harboring a malignant lead point is higher in patients >60 years with a previous history of malignancy or genetic risks for small bowel malignancy [6].

Concerning location, one of the largest studies, conducted in 1954 by Brayton et al., analyzed a total of 749 surgically diagnosed cases of intussusception occurring in 745 patients. According to their findings, 39% of cases were enteroenteric, 21% were ileocecal, 17% were colocolic, and 13% were ileocolic. The duodenum, stomach, or stomata was involved in about 10% of cases [2,9]. Additionally, a male to female ratio was found to be about 2:1 [2]; however, in another study, no gender predominance was observed [8]. To date, there are no studies with a large number of patients. More recently, a study conducted in 1971 reviewed 160 cases of intussusception and found that 24% of small bowel and 54% of colonic intussusceptions were of malignant origin [10]. In 1997, Azaretal conducted a retrospective review at the Massachusetts General Hospital with a total of 58 adult patients from 1964 until 1993, and he concluded that the diagnosis of intussusception was correctly done preoperatively in 32% of cases and was incorrect more often in small bowel benign intussusception which included 58 adult patients and concluded that intussusception was only correctly diagnosed preoperatively in 32% of cases, with small bowel intussusception of benign etiology the most frequently incorrectly diagnosed [10]. In a review by Hong et al., most lesions were located in the small bowel (49.5%), with most malignant tumors identified as metastatic carcinoma (48.7%), followed by lymphoma (26.2%) and GIST (21.3%). Colonic intussusception was less frequent (19.9%), with the main cause being adenocarcinoma (78.8%), followed by lymphoma (16.8%) and metastatic carcinoma (14.4%). In his review, adenocarcinoma was the most common cause of ileocolic intussusception (61.7%) [7].

Clinical presentation of these patients represents one of the major challenges since symptoms are usually non-specific in adult patients with intussusception, as in this case, where the initial presentation was shock. The lack of specificity of these findings adding to the low intussusception incidence in adults, leads to a broad list of differential diagnoses, but this should not delay intervention [2,8]. Pain is the most common symptom [2,8,10], present in 71-90% of cases [2]. Following pain, rectal bleeding, nausea, vomiting, and changes in bowel habits were common symptoms. Additionally, about 10% of cases had an abdominal mass noted on physical examination [2,8]. Symptoms are typically acute, lasting days to weeks, and the onset and duration of clinical symptoms are typically longer in large bowel than in small bowel intussusception [8]. Physical examination usually reveals a distended abdomen with decreased or absent bowel sounds, with variable tenderness to palpation and sometimes a palpable mass, findings that were also seen in the case reported [8]. If the patient presents the latter in their course, or the diagnosis is delayed, the physical examination may even exhibit signs of shock, cases in which peritonitis or bowel ischemia may be present, as in this patient [8].

Concerning laboratorial evaluation, elevations of C-reactive protein and leukocytosis are frequent [10] but non-significant unless ischemia or perforation have already developed [2]. In contrast, imaging evaluation is very often diagnostic, and in 36% of cases, the correct diagnosis can be made by ultrasound; by plain radiographs in 60% of cases, by barium enema in 36% of cases, and by CT scan in 72% of cases [2]. Not infrequently, the first imaging requested is an abdominal plain film, which may reveal unspecific signs of intestinal obstruction such as distended bowel loops without colonic gas or signs of perforation. Although this imaging modality may be useful, it lacks sensitivity and specificity for the diagnosis of intussusception [8]. Ultrasound is also an option, with the diagnosis made when the classic doughnut or target sign is demonstrated [2]. A more recent described variation of the latter is the crescent-in-doughnut sign consisting of concentric alternating hyperechoic and hypoechoic rings caused by the mucosal and muscularis layers of the bowels, and the submucosal layer, and the crescent being due to the involvement of mesentery as the intussusceptum [11]. Lastly, and surpassing the limitations of the previous modalities, CT offers the most sensitive imaging option [6], as previously described.

Flexible endoscopy, although allowing confirmation of the intussusception, its location, and biopsy, should be used with extreme caution in patients with acute obstruction due to a higher risk of perforation [6]. As soon as the diagnosis is made correctly, the discussion will revolve around the optimal treatment. Contrary to what is described for children, 70-90% of adults with intussusception will undergo surgery with resection as a definitive treatment [1].

Before the uplift of imaging diagnostic modalities, upfront surgery with laparotomy and resection was the standard of care for most surgeons [6]. Surgery was also the preferred approach due to the close association with malignancy as a lead point in the adult population; however, more recently, widespread use of CT has resulted in increased frequency of radiographic diagnosis of intussusception, sometimes asymptomatic. Regarding this fact, recent retrospective studies have demonstrated successful non-operative management of adult intussusceptions in >80% of patients [8].

The optimal treatment of intestinal intussusception in the adult population is still debated [3], and the most controversial matter is whether reduction should be performed [1,6]. Previous reports were in favor of resection without reduction due to the high possibility of malignancy without the ability to confirm the diagnosis in the preoperative period [3]. This approach to do an en-bloc resection of the affected intussuscepted segment was based on the potential risk of tumor cell embolization as well as the risk of perforation of the edematous, friable, or even ischemic bowel with consequent tumor cells and microorganisms seeding in the peritoneal cavity [1,3,4,6,8]. Nevertheless, this hypothesis still lacks high-quality evidence, as most come from case reports [4,6]. In those patients with small bowel intussusceptions in which a benign diagnosis is highly suspected, no signs of ischemia are found, or short gut syndrome is anticipated after resection, a reduction can be performed [1,3]. In contrast, in those cases in which malignancy is confirmed or suspected, all efforts should be made to perform a resection of the affected bowel in accordance with oncologic principles, including lymphadenectomy of the major draining vessels to allow proper staging and treatment [4,8].

Concerning the surgical approach, both laparotomy and laparoscopy can be done, depending on the experience of the surgeon [8]. The laparoscopic approach can be both diagnostic and therapeutic [6]. After resection of the affected bowel segment, primary enteroenteric, right-sided colocolic, or ileocolic anastomosis can be performed; as for resection for left-sided colic intussusception with associated obstruction, a Hartmann procedure is recommended, but primary anastomosis with or without a diverting ostomy can also be done [8].

In his review, Hong et al. found a postoperative morbidity rate of 22.1%, with surgical site infection being the most common complication. As for mortality, the rate was 5,2%, but most studies did not report detailed information [7]. In the same review, recurrence following treatment of adult intussusception was 6.5%, but due to the heterogenicity of follow-up in the reports, this information can be biased [7].

## Conclusions

Diagnosing an intussusception in an adult patient relying solely on the clinical presentation is a true clinical challenge. Due to the non-specific nature of the symptoms, many patients experience either a misdiagnosis or a delay in diagnosis. In fact, only half of the adult patients with intussusception are diagnosed preoperatively. Diagnosis relies on a high index of clinical suspicion and the aid of diagnostic imaging. The most challenging part of the management of patients presenting with intussusception is the ability to make an accurate diagnosis rather than treating the problem itself. In contrast to pediatric cases, adult cases tend to be due to pathological conditions, and potentially all forms of adult non-transient intussusceptions will require surgical treatment. Surgery is the mainstay of treatment in adult intussusception, and even if enteric intussusception can be managed by initial reduction followed by resection, colonic intussusception should be managed by upfront resection due to the high risk of an underlying malignant etiology. Idiopathic intussusception, though rare, can also be encountered, and a clear understanding of its pathophysiology and clinical presentation is an important aspect of surgical care. A full diagnostic evaluation and medical optimization should precede surgery in patients who lack a clear indication for emergency surgery.

In conclusion, adult intussusception is rare, the literature is scarce, and prospective studies with controls are lacking. It is possible to find reviews on adult intussusception, but there are only a few published systematic reviews with meta-analysis. This manuscript aims to provide some more insight into a rare cause of intestinal obstruction, that is this form of intussusception in an adult. Further investigation will allow more efficient and effective medical and surgical care.
